# Tenogenically Induced Allogeneic Peripheral Blood Mesenchymal Stem Cells in Allogeneic Platelet-Rich Plasma: 2-Year Follow-up after Tendon or Ligament Treatment in Horses

**DOI:** 10.3389/fvets.2017.00158

**Published:** 2017-09-26

**Authors:** Charlotte Beerts, Marc Suls, Sarah Y. Broeckx, Bert Seys, Aurélie Vandenberghe, Jeroen Declercq, Luc Duchateau, Martin A. Vidal, Jan H. Spaas

**Affiliations:** ^1^Global Stem Cell Technology, ANACURA Group, Evergem, Belgium; ^2^Equine Veterinary Practice Dr. Suls, Nederweert, Netherlands; ^3^Veterinary Practitioner, Oudenaarde, Belgium; ^4^Faculty of Veterinary Medicine, Department of Comparative Physiology and Biometrics, Ghent University, Merelbeke, Belgium; ^5^Cave Creek Equine Surgical & Imaging Center, Phoenix, AZ, United States

**Keywords:** mesenchymal stem cells, tendon, ligament, tenogenic, horse, peripheral blood

## Abstract

Poor healing of tendon and ligament lesions often results in early retirement of sport horses. Therefore, regenerative therapies are being explored as potentially promising treatment for these injuries. In this study, an intralesional injection was performed with allogeneic tenogenically induced mesenchymal stem cells and platelet-rich plasma 5–6 days after diagnosis of suspensory ligament (SL) (*n* = 68) or superficial digital flexor tendon (SDFT) (*n* = 36) lesion. Clinical, lameness and ultrasonographic evaluation was performed at 6 and 12 weeks. Moreover, a survey was performed 12 and 24 months after treatment to determine how many horses were competing at original level and how many were re-injured. At 6 weeks, 88.2% of SL (*n* = 68) and 97.3% of SDFT lesions (*n* = 36) demonstrated moderate ultrasonographic improvement. At 12 weeks, 93.1% of SL (*n* = 29) and 95.5% of SDFT lesions (*n* = 22) improved convincingly. Moreover, lameness was abolished in 78.6% of SL (*n* = 28) and 85.7% (*n* = 7) of SDFT horses at 12 weeks. After 12 months (*n* = 92), 11.8% of SL and 12.5% of SDFT horses were re-injured, whereas 83.8 of SL and 79.2% of SDFT returned to previous performance level. At 24 months (*n* = 89) after treatment, 82.4 (SL) and 85.7% (SDFT) of the horses returned to previous level of performance. A meta-analysis was performed on relevant published evidence evaluating re-injury 24 months after stem cell-based [17.6% of the SL and 14.3% of the SDFT group (*n* = 89)] versus conventional therapies. Cell therapies resulted in a significantly lower re-injury rate of 18% [95% confidence interval (CI), 0.11–0.25] 2 years after treatment compared to the 44% re-injury rate with conventional treatments (95% CI, 0.37–0.51) based on literature data (*P* < 0.0001).

## Introduction

Tendon and ligament lesions are very common injuries in sport and pleasure horses and often result in early retirement. The most frequently affected tendon in horses is the superficial digital flexor tendon (SDFT) (1). However, the type and frequency of tendon or ligament injuries depends on the discipline and the competitive level of the horse. Indeed, show jumping horses have a higher risk for SDFT tendinitis in the forelimb, whereas dressage horses are more subjected to hind limb suspensory ligament (SL) desmitis (1, 2).

The healing process of tendon and ligaments can be divided into four stages: an acute inflammatory phase, a subacute reparative phase, a collagen phase, and a chronic remodeling phase (3). It has been hypothesized that the most indicated moment for treatment is the transition between the inflammatory phase and the subacute reparative phase (3, 4), which is characterized by scar tissue production and collagen type III deposition by the fibroblasts. This tissue is weaker and less elastic than tendon tissue, which increases the risk of re-injury of the lesion site (3–8). The fibroplasia usually begins around the seventh day after the initial lesion, and therefore, the most adequate time of injection with stem cells would be around the fifth or the sixth day after injury (9–11).

Over the past years, tendon treatments with stem cells from different sources have been reported. Carvalho et al. described the use of autologous adipose tissue-derived mesenchymal stem cells (MSCs) suspended in a platelet concentrate for the treatment of induced SDFT tendinitis during a randomized controlled trial in eight mixed breed horses. Compared to the control group, a prevention of lesion progression, a better organization of collagen, and less inflammation were observed in the treatment group (12). Bone marrow (BM) is another frequently used source for MSCs. Smith et al. demonstrated favorable effects of autologous BM-derived MSCs on tendon healing in naturally occurring SDFT tendinitis in 12 horses (13). More precisely, the healed tendon of the horses in the treated group showed less stiffness and a better histological organization than the saline control group and reflected a trend to normalization of the tendon structure, which was significantly better than the saline control group. Despite these promising outcomes, the use of the horse’s own (autologous) MSCs is not always feasible in the field, and allogeneic MSCs can offer a valuable and more practical alternative. Indeed, allogeneic stem cells have been described for the treatment of different types of orthopedic injuries. Van Loon et al. described beneficial results after allogeneic umbilical cord blood (UCB)-derived MSC treatment of tendon and ligament injuries in 52 horses, and more precisely 77% (40 horses) regained their initial or a higher level of performance at 6 months after treatment than before the injury (14). In 2013, Ricco et al. described the use of allogeneic adipose tissue-derived MSCs combined with platelet-rich plasma (PRP) for the treatment of SDFT tendinitis in 19 horses. In this study, 89.5% of the horses returned to their previous competing level after a follow-up of 24 months (15).

Multipotent MSCs have been reported to cause ectopic bone formation after their transplantation in rabbit tendons (16, 17). However, to date, no ectopic tissue formation after MSCs injections has been described in horses. Fahy et al. suggested the use of predifferentiated MSCs toward the chondrocyte lineage to avoid inhibition of MSC chondrogenic differentiation by synovial fluid, factors, and macrophages present in the synovium of osteoarthritic joints (18). The safe use of tenogenically induced allogeneic MSCs in combination with PRP has been reported before in horses with SDFT and SL lesions (19). Moreover, a recent study of 20 horses with degenerative joint disease examined the clinical outcome after treatment with PRP alone, MSCs alone, or a combination of both PRP and MSCs. The latter pointed out that the combination of PRP + MSCs demonstrate significantly better clinical outcomes at short- and long-term in comparison with single treatments (6 weeks to 12 months) (20). However, long-term efficacy of a combination therapy of PRP and MSCs to treat tendon and ligament lesions in a large group of animals is indispensable from a clinical point of view.

Finally, the timing of the intralesional injection with MSCs depends on the source of MSCs. Autologous MSCs require a 2- to 3-week period of culture before implantation in the lesion, possibly compromising the treatment outcome. Currently, the time between harvesting and injection varies from 7 to 45 days (12). The use of allogeneic stem cells gives the possibility to prepare and characterize MSCs in advance and thus having stem cells available at any time.

The aim of our study is to evaluate the combined use of tenogenically induced MSCs and PRP in a large group of horses with tendon and ligament injuries. On the basis of preliminary data, we hypothesized that this combination for the treatment of SDFT tendinitis and SL desmitis would be safe and effective in comparison with reported conservative treatments.

## Materials and Methods

### Clinical Case Selection

In this study, 68 horses with SL lesions and 36 horses with SDFT tendinitis were included. A total of 104 dressage and show jumping horses were ultrasonographically and clinically examined before entering in the study in two different veterinary practices. A 7.5-MHz linear ultrasound probe was used to evaluate the injured region. A complete examination of the SDFT or SL was performed during each ultrasound examination with both transverse and longitudinal scans. The scoring system for evaluating ultrasound images and clinical soundness was adapted from the previous study by Beerts et al. (6) and can be found in Table 1.

**Table 1:** Different scoring systems used to evaluate SL and SDFT lesion healing.

A score
0 = 0% improvement or no ultrasonographic improvement
= Anechoic lesion + absence of fiber alignment
1 = 20% improvement or minor ultrasonographic improvement
= Hypoechoic areas/diffuse decrease in echogenicity + lack of parallel pattern
2 = 40% improvement or modest ultrasonographic improvement
= Multiple areas with decreased echogenicity + increased parallel fiber pattern
3 = 60% improvement or moderate ultrasonographic improvement
= Demarcation with healthy tissue less distinct + increased parallel fiber pattern
4 = 80% improvement or convincing ultrasonographic improvement
= Hardly any demarcation with healthy tissue + close to total fiber alignment
5 = 100% improvement or no ultrasonographic abnormalities
= Normal echogenicity + (almost) normal parallel fiber alignment

**B score**

0 = Lameness not perceptible under any circumstances
1 = Lameness is difficult to observe and is not consistently apparent, regardless of circumstances (e.g., under saddle, circling, inclines, hard surface)
2 = Lameness is difficult to observe at a walk or when trotting in a straight line but consistently apparent under certain circumstances
3 = Lameness is consistently observable at a trot under all circumstances
4 = Lameness is obvious at a walk
5 = Lameness produces minimal weight bearing in motion and/or at rest or a complete inability to move

**C score**

00 = Failure to return to work/re-injury
0 = Rehabilitating
1 = Return to work
2 = Return to previous level of work

Only horses with a lameness of one to three of five according to the American Association of Equine Practitioners (AAEP) grading system were allowed to participate in the study. Moreover, ultrasound imaging had to identify a clear core lesion present in the SL or SDFT, and no other lesions at other locations were allowed in this study. The lesion of the SL had to be restricted to one of the branches or to the origin.

Approval of the ethics committee was obtained (EC_2012_001) for blood sampling of the donor horse. The Federal Public Service of Health approved the Ethics Committee and granted the company with a laboratory recognition number (LA1700607), allowing the housing and handling of experimental animals providing blood for the isolation of stem cells.

The owners were informed about the study and gave their consent for their horses participating in the study.

### MSC Isolation, Tenogenic Induction, and PRP Preparation

Both tenogenically induced MSCs and PRP were prepared from peripheral blood (PB) from a 6-year-old German Warmblood horse according to good manufacturing practices and characterized as previously described (21–23).

Briefly, 20 ml PB was collected aseptically from the *vena jugularis* in ethylenediaminetetraacetic acid (EDTA) tubes for MSC isolation from one donor. Subsequently, the blood was centrifuged, and the buffy coat was collected for gradient centrifugation. After washing, 20 million peripheral blood mononuclear cells were seeded per T_75_ flask and cultured until 60% confluency was reached corresponding with 0.18–0.32 million MSCs. Trypsinization was performed with 0.25% trypsin-EDTA until passage 5 (P5) was reached. All batches between P6 and P8 were seeded in T75 flasks and tenogenically induced by supplementing tendogenic growth factors to the expansion medium until 80% confluency was reached and tenogenic differentiation markers were upregulated (19, 21, 22). A higher passage was used to allow upscaling in future cell manufacturing processes, which is considered to be possible thanks to the phenotypic stability of PB-MSCs from P5 up to P10 (21, 23, 24). After trypsinization, all the cells were resuspended in 1 ml DMEM low glucose (Life Technologies) with 10% of dimethyl sulfoxide (Sigma). The samples were stored at −80°C until all quality controls [sterility testing; mycoplasma testing; endotoxine testing; flow cytometry for CD29, CD44, and major histocompatibility complex (MHC) II; and viability staining] were completed.

For PRP preparation, 300 ml PB was taken in a citrate phosphate dextrose adenine-1 single blood bag (Terumo^®^). Platelets were purified by means of subsequent centrifugation steps as previously reported (25) until more than 80% platelets were obtained at a concentration of more than 100 × 10^6^ platelets in 1 ml plasma. The leukocyte count of PRP was 0.5% (<100 leukocytes/μl). Different batches of PRP were necessary to produce sufficient excipient for all the treatments. Allogeneic cells and allogeneic PRP were shipped on dry ice for clinical application.

### Treatment

**Figure 1:**
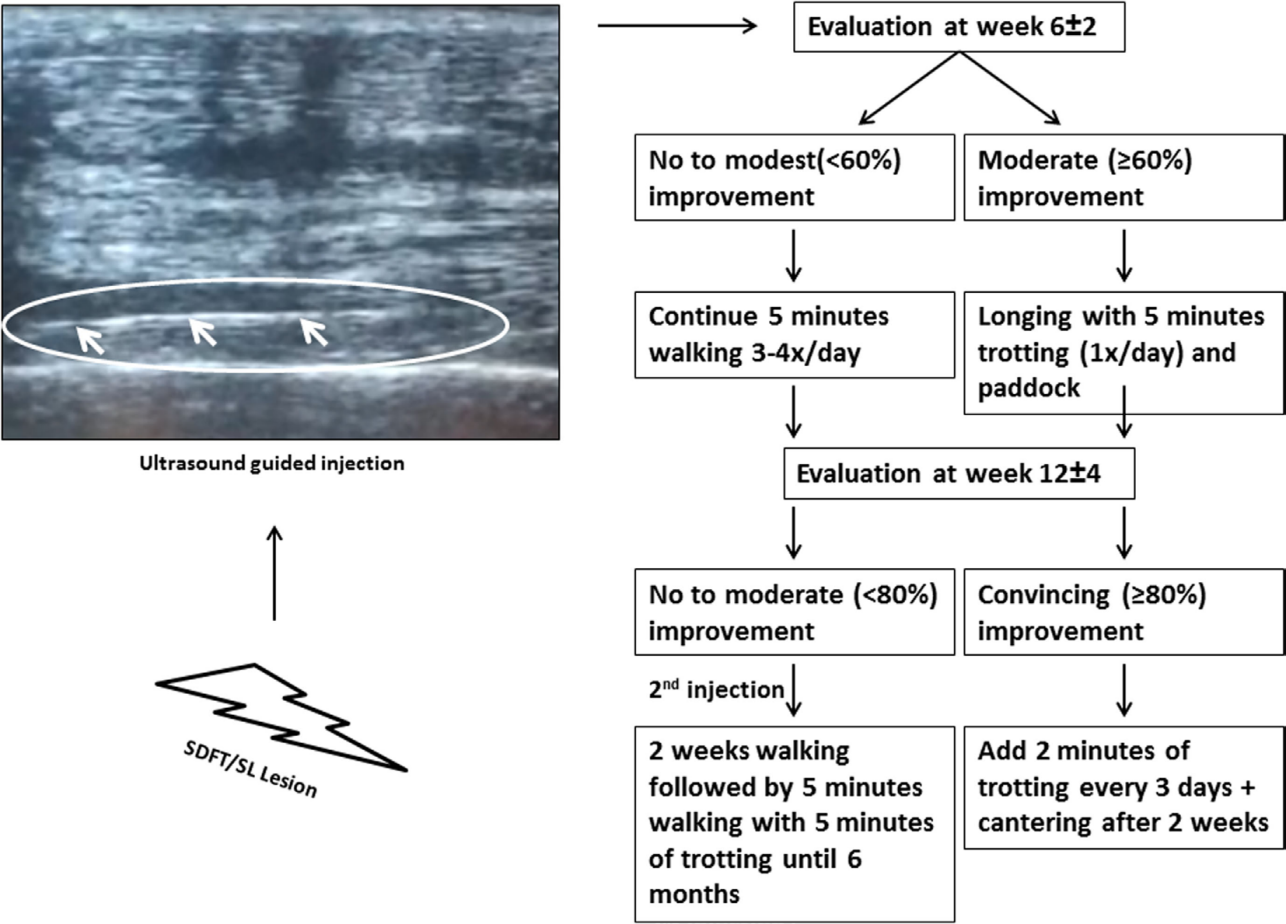
The ultrasound image indicates the needle inserted for injection. A standardized rehabilitation program was applied as strictly as possible starting from the evaluation at 6 ± 2 weeks (Exam 1).

The horses were injected approximately 5–6 days after the lesion occurred. When the diagnosis of SL or SDFT lesion was established, an intravenous injection of a sedative agent was performed, and the injection site was thoroughly cleaned and disinfected. For all of the 104 patients included in this study, an intralesional ultrasound-guided injection (Figure 1) with allogeneic tenogenically induced MSCs and PRP was performed by an equine orthopedic specialist. After thawing, 1 ml of PRP (containing between 100,000,000 and 150,000,000 platelets per treatment) and 1 ml of tenogenically induced PB-derived MSCs (with the number of stem cells ranging from 2,000,000 to 3,000,000 with a viability of at least 50% due to storage and frozen transport) were mixed in one syringe before the ultrasound-guided intralesional injection. The same dose was used for all lesion sizes included in the study. For the next 3–5 days, horses were administered oral non-steroidal anti-inflammatory drugs (NSAIDs) consisting of 1–2 g phenylbutazone.

### Evaluation Protocol

The same independent veterinarians (MS, BS, and AV) were asked to report the results of the examination of each horse in a standardized follow-up document as demonstrated in Table 1. First, an “A score” regarding the clinical and ultrasonographic improvement had to be given to all the horses by using the initial ultrasound images as comparison. This score ranges from 0 to 5 (0 meaning no ultrasonographic improvement and 5 meaning 100% ultrasonographic improvement) and is an addition of two previously described ultrasonographic grading scales, more precisely an echogenicity scale (0 meaning anechoic lesion and 5 meaning normal echogenicity) and fiber arrangement scale (0 meaning absence of fiber alignment and 5 meaning normal parallel fiber alignment) (26). For a number of these horses, a “B score,” relating to the lameness evaluation [following the AAEP grading system (20)], and a “C score,” regarding the allowed level of exercise, were also given (Table 1). Unfortunately, not all veterinarians recorded a “B and C score” for the examined horses, so the quantity of these scores is lower than the quantity of “A scores” recorded. This scoring system was considered an objective manner in which to report the data.

### Follow-up and Rehabilitation

The first week following the treatment, all treated horses were clinically examined daily by a veterinarian, and more precisely they were examined for lameness at a walk and pain, heat, and swelling at the injection site. All horses had to walk for 5 min three to four times a day until the first scoring evaluation of the veterinarian at week 6 ± 2 (Exam 1). A clinical evaluation, lameness assessment, and ultrasonography were performed. If the healing progressed successfully, the amount of exercise of the horse could be increased (an A score of 3 = at least 60% improvement). On the other hand, if there was no significant improvement, the animal had to continue walking. A second examination was performed in 51 (29 SL and 22 SDFT) of the 104 horses at week 12 ± 4 (Exam 2). Unfortunately, a number of owners failed to present their horse for the second examination, which reduces the number of scores collected at that time point. Again, depending on the degree of improvement (denoted by an A score of 4 = at least 80% improvement), an increase in exercise was allowed. More details on the rehabilitation program can be found in Figure 1. Horses that showed moderate or less improvement (denoted by an A score of 3 or less = 60% improvement or moderate ultrasonographic improvement) received a second injection of tenogenically induced PB-derived MSCs in combination with PRP at the second examination. During the first 2 weeks following injection, these horses were hand-walked for 5 min three to four times per day, and then they had to walk for 20 min and trotted for 5 min until 6 months after the initial injection. After 6 months, the horses were allowed to go back into full work. Furthermore, at 12 (exam 3) and 24 months (exam 4) after treatment, a survey was performed to determine how many horses were competing at their original level again. More precisely, the owners were asked if the horses were competing at the same level again. If the horse was not competing, the owners were asked for the reason for retirement (re-injury, other reason). The outcome of 68 horses with SL lesions and 24 horses with SDFT lesions could be obtained at 12 months (*n* = 92) after treatment and of 68 horses with SL lesions and 21 horses with SDFT lesions after 24 months (*n* = 89).

### Statistical Analysis

Percentage of re-injury was calculated by dividing the number of horses that relapsed and retired due to an injury of the treated SL or SDFT after 24 months by the total number of evaluated horses multiplied by 100. Horses that were not available for follow-up or retired due to other reasons were not included in this re-injury calculation. For the meta-analysis, the literature was first reviewed for publications on the treatment of similar equine tendon and ligament injuries, using search terms such as “horses,” “stem cells,” “allogeneic,” “autologous,” “PRP,” “SL,” and “SDFT” and in the Web of Science database, PubMed, and Google search. All case studies consisting of stem cell-based therapies for SDFT and SL treatment were included in one group of the analysis. All clinical results of SDFT and SL treatments with medical standard of care were included in the second group. Furthermore, the re-injury rates were compared between different stem cell treatments (including this study) and conventional treatments using the random effects model and the Wald test. The meta-analysis results are presented in a forest plot.

## Results

### Follow-up and Rehabilitation

**Figure 2:**
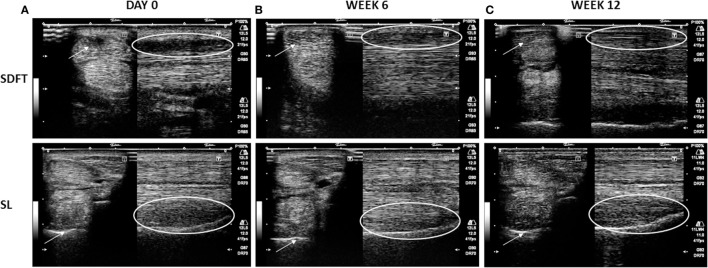
Representative transverse (arrows) and longitudinal (circles) images of superficial digital flexor tendon (SDFT) (1) and suspensory ligament (SL) (2) at the moment of acute lesion (day 0) **(A)**, at 6 weeks after treatment **(B)** with an A score of 3 (at least 60% improvement), and at 12 weeks after treatment **(C)** with an A score of at least 4 (at least 80% of improvement). The arrows and circles indicate the location of the lesion.

Following the injection, none of the horses showed any adverse reactions. NSAIDs were administered for 3–5 days and might have reduced any possible swelling due to the injection. At 6 ± 2 weeks (Exam 1), 88.2% of the horses with SL lesions (*n* = 68) received an A score of at least 3 (at least 60% improvement). In the group of horses suffering from SDFT tendinitis, 98.2% received an A score of at least 3 (*n* = 36) (Figure 2). Of the 104 horses with SL or SDFT lesion, 42 horses received a B score and 45 horses received a C score at Exam 1. Regarding the B score, 82.3% of the SL group and 87.5% of the SDFT group presented no or very light lameness (score 0–1). This demonstrated an early clinical improvement in most of the horses. As prescribed by the veterinarian, 75% of horses with SL lesion and 88.9% of horses with SDFT tendinitis were rehabilitating (C score of 0) at Exam 1 (Table 2).

**Table 2:** Percentage of treated patients with corresponding A, B, and C scores at a 6 ± 2 weeks (Exam 1) (*n*_1_) and 12 ± 4 weeks (Exam 2) (*n*_2_) and with C scores at 12 (Exam 3) and 24 months (Exam 4) after treatment of the suspensory ligament (SL) or superficial digital flexor tendon (SDFT) with allogeneic tenogenically induced mesenchymal stem cells in combination with platelet-rich plasma.

A score (%)			0	1	2	3	4	5
SL	Exam 1	*n*_1_ = 68	0	0	11.8	29.4	44.1	14.7
	Exam 2	*n*_2_ = 29	0	0	0	6.9	41.4	51.7
SDFT	Exam 1	*n*_1_ = 36	0	0	2.8	30.6	52.8	13.9
	Exam 2	*n*_2_ = 22	0	0	0	4.6	45.5	50.0

**B score (%)**			**0**	**1**	**2**	**3**	**4**	**5**

SL	Exam 1	*n*_1_ = 34	35.3	47.1	17.7	0	0	0
	Exam 2	*n*_2_ = 28	78.6	14.3	3.6	3.6	0	0
SDFT	Exam 1	*n*_1_ = 8	50.0	37.5	12.5	0	0	0
	Exam 2	*n*_2_ = 7	85.7	0	14.3	0	0	0

**C score (%)**			**00**	**0**	**1**	**2**		

SL	Exam 1	*n*_1_ = 36	0	75.0	22.2	2.8		
	Exam 2	*n*_2_ = 28	0	42.9	35.7	21.4		
	Exam 3	*n*_3_ = 68	11.8	2.9	1.5	83.8		
	Exam 4	*n*_4_ = 68	17.6	0	0	82.4		
SDFT	Exam 1	n_1_ = 9	0	88.9	11.1	0		
	Exam 2	*n*_2_ = 7	0	28.6	71.4	0		
	Exam 3	*n*_3_ = 24	12.5	8.3	0	79.2		
	Exam 4	*n*_4_ = 21	14.3	0	0	85.7		

A total of 51 horses (*n* = 29 horses with SL lesion; *n* = 22 horses with SDFT tendinitis) were subjected to an examination at 12 ± 4 weeks (Exam 2), In the SL group, 41.4% of the horses received an A score of 4 and 51.7% an A score of 5 (score 4 + 5 = 93.1%). In the SDFT group, similar A scores were observed in 45.5 and 50.0% of the horses (score 4 + 5 = 95.5%), respectively (Figure 2). The AAEP lameness evaluation or B score and the C score were documented in 28 horses with SL lesion and 7 horses with SDFT tendinitis. In 78.6% of the horses with SL lesion and 85.7% of the horses with SDFT tendinitis, no more lameness was observed (score 0) at Exam 2. Finally, 57.1 (= 35.7 + 21.4) and 71.4% of the horses returned to work in SL group (score 1 + 2) and SDFT group, respectively (Table 2) at 12 weeks (Exam 2).

At 12 months after treatment, 83.8% (*n* = 57) of SL horses (*n* = 68) were competing at initial level and 11.8% (*n* = 8) were re-injured, whereas 79.2% (*n* = 19) of SDFT horses (*n* = 24) returned to previous level and 12.5% (*n* = 3) were re-injured. After 24 months, 82.4% (*n* = 56) of the horses with SL lesions (*n* = 68) were competing at their initial level; however, 22% (*n* = 15) of them [horses who received an A score of 3 or less at 12 ± 4 weeks (Exam 2)] received a second injection at Exam 2 with tenogenically induced PB-derived MSCs before getting to previous level. Moreover, 14.7% (*n* = 10) of the horses in the SL group relapsed and were retired, and finally, 2.9% (*n* = 2) of the horses had to quit sport for multiple reasons. In the second group (SDFT, *n* = 21), 85.7% (*n* = 18) of the horses were competing at their initial level after 24 months, yet 33.3% (*n* = 7) of the competing horses received a second injection and 14.3% (*n* = 3) of the horses with SDFT lesions were retired after a relapse.

### Meta-analysis

**Figure 3:**
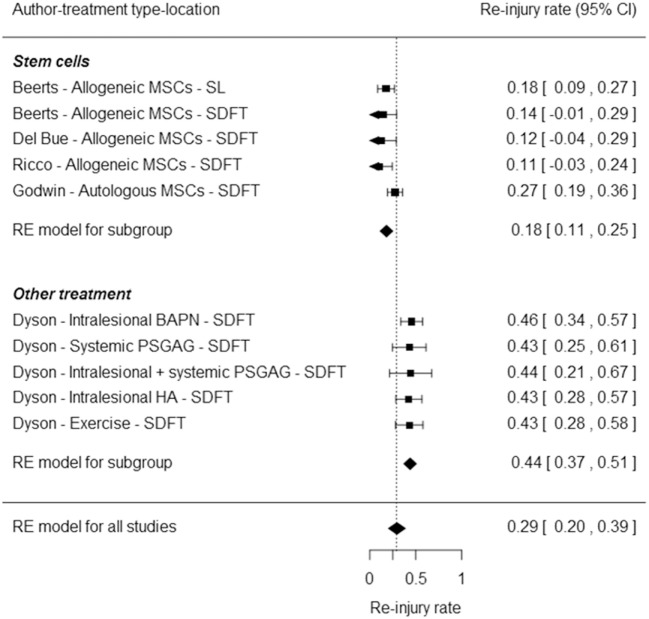
Forest plot representing a comparison of the re-injury rates between different stem cell treatments from the current study (Beerts) and other studies (15, 27, 28) with conventional treatments (29) using a meta-analytic approach based on the random effects model. The squares represent the re-injury rates of individual studies, with the whiskers corresponding to the 95% confidence interval (CI). The diamonds correspond to the re-injury rates and 95% CI of the two subgroups and overall.

The meta-analysis based on the reported stem cell studies including this study revealed a highly significant difference between stem cell treatment and other treatments (*P* < 0.0001). The stem cell-treated group has a significantly lower re-injury rate equal to 18% [95% confidence interval (CI), 0.11–0.25] compared to the conventional treatments with a re-injury rate equal to 44% (95% CI, 0.37–0.51). The odds of re-injury is significantly smaller in the stem cell-treated group compared to the conventional treatment group (OR = 0.78, 95% CI, 0.71–0.86, *P* < 0.0001). The results are shown in the forest plot in Figure 3.

## Discussion

In this study, 68 horses with SL lesions and 36 horses with SDFT tendinitis were treated with tenogenically induced PB-derived MSCs combined to PRP. No adverse reactions could be observed in any of the horses; however, it should be emphasized that NSAIDs were provided for 3–5 days after the intralesional injection to assist in reducing the originally present swelling and pain. Nevertheless, it has been previously reported that allogeneic MSCs do not induce an immune response and associated discomfort in the receiver horses. Indeed, in a previous study, no significant immune reaction was reported in the SL and SDFT of 25 horses after intralesional treatment with tenogenically induced PB-derived MSCs without administering NSAIDs (19). Moreover, no local adverse reaction was reported after allogeneic tenogenically induced MSC treatment of four horses with desmitis of the accessory ligament of the deep digital flexor tendon (6). Although the latter horses were treated with NSAIDs for 5 days before the injection with MSCs, no more NSAIDs were administered after treatment. In another study, 19 horses affected by SDFT tendinitis were treated with an association of allogeneic adipose tissue-derived MSCs and PRP without the administration of NSAIDs, and again, there was no proof of any local immunological reaction (absence of heat, swelling, pain, or lameness) in the treated horses (15). A study comparing the effects of autologous mesenchymal progenitor cells (MPCs), allogeneic MPCs, or BM supernatant alone on induced SDFT tendinitis could not show differences in cell-mediated immune responses between different stem cell sources. The horses received a single intravenous injection of NSAIDs before the induction of the lesion and antibiotics for several days (30). Finally, Carrade et al. reported a similar response in the joint following an intraarticular injection of allogeneic MSCs compared to autologous MSCs (31).

All the aforementioned work indicates that there do not appear to be remarkable differences in safety between autologous and allogeneic MSCs. It is possible that MSCs may have the ability to restrain immune cells and to reduce the production of pro-inflammatory cytokines (32, 33). However, repeated intravenous injections of allogeneic equine MSCs have been shown to result in elevated numbers of circulating CD8^+^ T cells, suggesting a mild alloantigen-directed cytotoxic response (34). Nevertheless, there was no indication for any organ toxicity or systemic inflammatory response, and another study also reported no clinical issues after single or repeated intravenous injections in 291 horses (35). Also in this study, no adverse event was noticed after a second injection in 15 horses with SL and 7 horses with SDFT lesions. A recent study reported elevated synovial total nucleated cell counts after repeated intraarticular injections of allogeneic BM-derived MSCs (36). However, the clinical relevance remains to be demonstrated (37) because allogeneic MSCs would not enhance a hypersensitivity response according to other researchers (38). On the other hand, Owens et al. reported the presence of anti-MSCs antibodies in the serum of 37% of horses injected with allogeneic BM- or adipose tissue-derived MSCS. Currently, the significance of these antibodies is unknown and warrants further research in case of repeated injections (39). In addition, an *in vitro* study described a cytotoxic antibody response in recipient horses after the injection of allogeneic MHC-mismatched BM-derived MSCs (40). This indicates that MHC levels are crucial for allogeneic transplantations and warrant analyses before clinical use in horses. In a study investigating the antibody response to allogeneic MSC transplantation after an intradermal injection of allogeneic MSCs into the neck of six horses, Pezzanite et al. described the capacity of allogeneic MSCs to elicit antibody responses (41). In this study, we used a considerably lower amount of MSCs (2–3 × 10^6^ MSCs per ml) compared to other studies [10 × 10^6^ MSCs per ml (36), 25–80 × 10^6^ MSCs per injection (39), and 30 × 10^6^ or 50 × 10^6^ MSCs per injection (41)]. This could explain the low immunogenicity and the absence of observable adverse events in the horses that were injected in this study. Finally, Berglund et al. described the downregulation of MHC I and MHC II on equine BM-derived MSCs without alteration of other phenotypic cell surface markers when culturing the cells in specific media, suggesting that media selection could be an interesting option to guaranty the safe use of allogeneic MSCs (42). Nevertheless, more robust *in vivo* studies are required to detail the precise immunologic effect of allogeneic cell treatment in the horse.

The PRP used in our study is leukocyte-poor (LP) PRP. Nevertheless, to date, the optimal combination of platelet and leukocytes is not known. However, different *in vitro* studies suggested a lower expression of pro-inflammatory cytokines after the intratendinous injection of leukocyte-reduced PRP compared to leukocyte-rich PRP (43, 44). Furthermore, persistent inflammation of the tendon engenders more scar tissue formation resulting in inferior tendon repair (44). Another study described improved functional outcome scores after the injection of LP-PRP in the treatment of knee osteoarthritis in human when compared to hyaluronic acid or placebo. This difference was not observed after the injection of leukocyte-rich PRP (45).

At 6 and 12 weeks, the clinical and ultrasonographic improvements of more than 88% of the treated horses with SL lesions or SDFT tendinitis seemed promising. Nevertheless, a quantitative ultrasonographic and lameness assessment would have provided a more objective tendon evaluation. After 12 months (*n* = 92), 83.8% of SL and 79.2% of SDFT horses returned to previous level of performance. Finally, 24 months after treatment (*n* = 89), 82.4% of the horses with SL lesions and 85.7% of the horses with SDFT lesions were competing at their initial level. These results are comparable, yet a bit lower than those obtained by Ricco et al. on 19 horses with SDFT tendinitis (89.5% to original level after 24 months) (15), by Del Bue et al. on 16 horses with SDFT tendinitis (87.5% back to work at 23 weeks) (27), and finally by Toricelli et al. on 13 horses with SDFT and SL lesions (84.6% back to competition after 12 months) (46). In our study, 22 and 33.3% of the horses affected with SL lesions and SDFT tendinitis, respectively, did also require a second treatment at 12 ± 4 weeks (Exam 2) before returning to the original competing level. However, these horses were all high-level athletes, and therefore, the lower return to previous level and second treatments were perhaps not surprising. Van Loon et al. reported the necessity of a second injection of allogeneic UCB MSCs in 4.3% of the 23 horses with SDFT lesions and 18.2% of the 22 horses with SL lesions (14). Although this is considerably lower than the results in this study, it should be mentioned that the re-injury rate was considered at 6 months after treatment only, and no results are available at 24 months, which would have provided valuable comparative data.

In this study, 11.8% of SL and 12.5% of SDFT horses were re-injured after 12 months (*n* = 92) and 17.6% and 14.3% were re-injured at 24 months, respectively (*n* = 89). Unfortunately, tendon and ligament injuries have a rather poor prognosis with conventional treatment. The meta-analysis demonstrated a significantly lower re-injury rate of 18% (95% CI, 0.11–0.25) in the stem cell-treated group compared to conventional treatments with a re-injury rate of 44% (95% CI, 0.37–0.51) (*P* < 0.001). Different non-blinded case series with different stem cell sources for both tendon (SDFT) and ligament (SL) lesions have been included in the meta-analysis. This has a lower conclusive value in comparison with a placebo-controlled, double-blinded clinical trial with all animals treated and rehabilitated under the same circumstances with the possibility of a direct comparison between different treatment groups. Nevertheless, overall results from conservative treatment studies demonstrate considerably higher re-injury rates than all stem cell studies considered for the meta-analysis. Indeed, in one study, the re-injury rate of proximal SL desmitis was 46% in jumping horses and 37% in dressage horses (47). Furthermore, a two-part study conducted by Dyson on horses affected with SDFT tendinitis resulted in a re-injury rate after 2 years ranging from 42.5 to 44.4% in a first group of 135 horses treated conservatively or medically. In the second group of 68 horses treated with beta aminopropionitrile fumarate, 16% of the horses relapsed in the treated limb, but, when the uninjured limb was taken into consideration, the re-injury rate was up to 45.6%, which is comparable to the re-injury rate reported in the first group (29).

In this study, the MSCs were combined with PRP for its capacity to provide growth factors, which potentiate the early healing of the lesion by stimulating angiogenesis and by enhancing MSC proliferation (27). Indeed, beneficial clinical effects of the combination of PRP and MSCs in comparison to MSCs alone have been described multiple times after treatment of tendon lesions with allogeneic adipose tissue-derived MSCs (15, 27) or BM mononucleated cells (27) combined with autologous PRP. Nevertheless, it should be emphasized that PRP alone may also have beneficial effects on tendon healing (48). This definitely warrants further investigation to determine whether the observed results in this study are due to the MSCs or PRP or the combination of both.

As previously mentioned, the timing of the intralesional injection depends on the source of MSCs (autologous versus allogeneic). In this study, the MSCs were injected 5–6 days after the initial lesion, more precisely after the acute inflammatory response but before the formation of fibrous tissue (3).

Even though the results of this study seem promising, the study has some limitations. First, it was not blinded, and there was no untreated or placebo-treated control group. However, considering it was not a preclinical study but an evaluation of a large group of sport horses treated by independent veterinarians, it would be difficult to convince the owners to include their animal in the placebo group. Nevertheless, future studies should include a group with at least best supportive care or PRP treatment alone as a control group. To put these data in a larger context and still make valuable comparisons, the aforementioned meta-analysis was performed comparing the re-injury rate of stem cell-based therapies (including our data) and conventional therapies. Our data comply with results from all other studies, and the stem cell-based therapies have a significantly lower re-injury rate than the conventional therapies. Nonetheless, predifferentiating MSCs remains a sensible strategy to reduce the risk of unwanted cell formation in an inflamed tendon environment as reported in rabbits before (16, 17) and to direct the cells toward the tissue that needs regeneration. Comparing the re-injury results of SDFT lesions in this study (14.3%) with the re-injury rate of 113 horses with SDFT lesions (27.4%) treated with undifferentiated MSCs without PRP (28), a considerable difference can be observed.

A second limitation is that all the horses were treated with a combined therapy of PRP and MSCs, which makes it impossible to evaluate the difference between the potential therapeutic effect of PRP in comparison to that of the tenogenically induced PB-derived MSCs. Therefore, future studies should focus on the comparison of predifferentiated with undifferentiated MSCs and investigate the effect of PRP addition on the clinical outcome. Another limitation is the difference in the rehabilitation programs between stables. Although a standardized rehabilitation program was given by the veterinarians to the owners to obtain comparable data, this program was adapted to each patient, and this is reflected in the C score. More precisely, in the SL group, 75% of the horses were rehabilitating, 22.2% returned to work, and 2.8% returned to previous level at 6 ± 2 weeks, whereas 42.9% were rehabilitating, 35.7% returned to work, and 21.4% returned to previous level at a 12 ± 4 weeks. In the SDFT group, 88.9% of the horses were rehabilitating and 11.1% returned to work at 6 ± 2 weeks, whereas at 12 ± 4 weeks, 28.6% were rehabilitating and 71.4% returned to work. These results suggest that the SL heals faster than the SDFT; however, they can also indicate that the initial SL lesions were less severe than those of the SDFT. Moreover, in this study, several horses dropped out for long-term evaluation. The main reason for this obstacle was a physical move of the patient due to a change of rider, owner, or veterinarian. Having all the horses for the long-term evaluation would have allowed us to draw more conclusions on the resistance to re-injury of the repaired tissue. Nevertheless, a large amount of horses could be identified at 12 (*n* = 98) and 24 (*n* = 89) months after treatment and provided substantial data on functional recovery.

## Conclusion

This study reports a safe and promising use of tenogenically induced allogeneic PB-derived MSCs in allogeneic PRP for the treatment of SL and SDFT lesions in 104 horses with statistically significant lower re-injury rate 2 years after treatment (*P* < 0.0001) in comparison to conventional therapies.

## Ethics Statement

Approval of the ethical committee was obtained (EC_2012_001) for blood sampling of the donor horse. The Federal Public Service of Health approved the Ethics Committee and granted the company with a laboratory recognition number (LA1700607), allowing the housing and handling of experimental animals providing blood for the isolation of stem cells. The ethics committee specifically approved this study.

## Author Contributions

CB: data analysis, preparation manuscript, study design, drafting article, and final approval. MS: study execution, data analysis, study design, and final approval. SB: preparation manuscript, study design, drafting article, and final approval. BS, AV, and JD: study execution, data analysis, and final approval. LD: data analysis and interpretation, study design, drafting article, and final approval. MV: study design, drafting article, and final approval. JS: data analysis, study design, drafting article, and final approval.

## Conflict of Interest Statement

The author JS declares competing financial interests as shareholder in Global Stem cell Technology (GST). CB, SB, and JS are employed by GST and SB and JHS are inventors of several (pending) patents owned by GST (BE2012/0656, PCT/EP2013/070247, PCT/EP2013/070257, and PCT/EP2013/075782). The content of this manuscript contains a product under development owned by GST. The other authors declare that the research was conducted in the absence of any commercial or financial relationships that could be construed as a potential conflict of interest.

## References

[1] Thorpe CT Clegg PD Birch HL. A review of tendon injury: why is the equine superficial digital flexor tendon most at risk? Equine Vet J (2010) 42:174–80. 10.2746/042516409X480395 20156256

[2] Murray RC Dyson SJ Tranquille C Adams V. Association of type of sport and performance level with anatomical site of orthopaedic injury diagnosis. Equine Vet J (2006) 36:411–6. 10.1111/j.2042-3306.2006.tb05578.x 17402457

[3] Spaas JH Guest DJ Van de Walle GR. Tendon regeneration in human and equine athletes: Ubi Sumus-Quo Vadimus (where are we and where are we going to)? Sports Med (2012) 42:871–90. 10.2165/11635390-000000000-00000 22963225

[4] Richardson CE Dudhia J Clegg PD Smith R Stem cells in veterinary medicine: attempts at regenerating equine tendon after injury. Trends Biotechnol (2007) 25:409–16. 10.1016/j.tibtech.2007.07.009 17692415

[5] Baxter GM Tendon and ligament injuries and disease. In: Baxter GM, editor. Adams and Stashak’s Lameness in Horses. Chichester, FL: Wiley-Blackwell (2012). p. 927–38.

[6] Beerts C Seifert C Zimmerman M Felix E Suls M Mariën T Desmitis of the accessory ligament of the equine deep digital flexor tendon: a regenerative approach. J Tissue Sci Eng (2013) 4:1–7. 10.4172/2157-7552.1000125

[7] Dakin SG Dudhia J Smith RKW. Resolving an inflammatory concept: the importance of inflammation and resolution in tendinopathy. Vet Immunol Imunopathol (2014) 158:121–7. 10.1016/j.vetimm.2014.01.007 24556326 PMC3991845

[8] James R Kesturu G Balian G Chhabra B. Tendon: biology, biomechanics, repair, growth factors, and evolving treatment options. J Hand Surg Am (2008) 33:102–12. 10.1016/j.jhsa.2007.09.007 18261674

[9] McCullagh KG Goodship AE Silver IA Tendon injuries and their treatment in the horse. Vet Rec (1979) 105:54–7. 10.1136/vr.105.3.54 400209

[10] Obaid H Connell D. Cell therapy in tendon disorders: what is the current evidence? Am J Sports Med (2010) 38:2123–32. 10.1177/0363546510373574 20699425

[11] Williams IF Heaton A McCullagh KG. Cell morphology and collagen types in equine tendon scar. Res Vet Sci (1980) 28:302–10. 7414083

[12] Carvalho AM Badial PR Alvarez LEC Yamada AL Borges AS Deffune E Equine tendonitis therapy using mesenchymal stem cells and platelet concentrates: a randomized controlled trial. Stem Cell Res Ther (2013) 4:1–13. 10.1186/scrt236 23876512 PMC3854756

[13] Smith RK Werling NJ Dakin SG Alam R Goodship AE Dudhia J. Beneficial effects of autologous bone marrow-derived mesenchymal stem cells in naturally occurring tendinopathy. PLoS One (2013) 8:e75697. 10.1371/journal.pone.0075697 24086616 PMC3783421

[14] Van Loon VJ Scheffer CJ Genn HJ Hoogendoorn AC Greve JW Clinical follow-up of horses treated with allogeneic equine mesenchymal stem cells derived from umbilical cord blood for different tendon ad ligament disorders. Vet Q (2014) 34:92–7. 10.1080/01652176.2014.949390 25072527

[15] Ricco S Renzi S Del Bue M Conti V Merli E Ramoni R Allogeneic adipose tissue-derived mesenchymal stem cells in combination with platelet rich plasma are safe and effective in the therapy of superficial digital flexor tendonitis in the horse. Int J Immunopathol Pharmacol (2013) 26:61–8. 10.1177/03946320130260S108 24046950

[16] Awad HA Boivin GP Dressler MR Smith FN Young RG Butler DL. Repair of patellar tendon injuries using a cell-collagen composite. J Orthop Res (2003) 21:420–31. 10.1016/S0736-0266(02)00163-8 12706014

[17] Harris MT Butler DL Boivin GP Florer JB Schantz EJ Wenstrup RJ. Mesenchymal stem cells used for rabbit tendon repair can form ectopic bone and express alkaline phosphatase activity in constructs. J Orthop Res (2004) 22:998–1003. 10.1016/j.orthres.2004.02.012 15304271

[18] Fahy N de Vries-van Melle ML Lehmann J Wei W Grotenhuis N Farrell E Human osteoarthritic synovium impactes chondrogenic differentiation of mesenchymal stem cells via macrophage polarisation state. Osteoarthritis Cartilage (2014) 22:1167–75. 10.1016/j.joca.2014.05.021 24911520

[19] Broeckx S Zimmerman M Aerts D Seys B Suls M Mariën T Tenogenesis of equine peripheral blood-derived mesenchymal stem cells: in vitro versus in vivo. J Tissue Sci Eng (2012) 11:1–6. 10.4172/2157-7552.S11-001

[20] Broeckx S Zimmerman M Crocetti S Suls M Mariën T Ferguson SJ Regenerative therapies for equine degenerative joint disease: a preliminary study. PLoS One (2014) 9:e85917 10.1371/journal.pone.0085917 24465787 PMC3896436

[21] Gomiero C Bertolutti G Martinello T Van Bruaene N Broeckx SY Patruno M Tenogenic induction of equine mesenchymal stem cells by means of growth factors and low level laser therapy. Vet Res Commun (2016) 40:39–48. 10.1007/s11259-016-9652-y 26757735

[22] Spaas JH De Schauwer C Cornillie P Meyer E Van Soom A Van de Walle GR Culture and characterization of equine peripheral blood mesenchymal stromal cells. Vet J (2013) 195:107–13. 10.1016/j.tvjl.2012.05.006 22717781

[23] Spaas JH Broeckx SY Chiers K Ferguson SJ Casarosa M Van Bruaene N Chondrogenic priming at reduced cell density enhances cartilage adhesion of equine allogeneic MSCs – a loading sensitive phenomenon in an organ culture study with 180 explants. Cell Physiol Biochem (2015) 37:651–65. 10.1159/000430384 26344791

[24] Vandenberghe A Broeckx SY Beerts C Seys B Zimmerman M Verweire I Tenogenically induced allogeneic mesenchymal stem cells for the treatment of proximal suspensory ligament desmitis in a horse. Front Vet Sci (2015) 2:49. 10.3389/fvets.2015.00049 26664976 PMC4672201

[25] Araki J Jona M Eto H Aoi N Kato H Suga H Optimized preparation method of platelet-concentrated plasma and noncoagulating platelet-derived factor concentrates: maximization of platelet concentration and removal of fibrinogen. Tissue Eng Part C Methods (2012) 18:176–85. 10.1089/ten.TEC.2011.0308 21951067 PMC3285602

[26] Marr CM McMillan I Boyd JS Wright NG Murray M. Ultrasonographic and histopathological findings in equine superficial digital flexor tendon injury. Equine Vet J (1993) 25:23–9. 10.1111/j.2042-3306.1993.tb02896.x 8422880

[27] Del Bue M Ricco S Ramoni R Conti V Gnudi G Grolli S Equine adipose-tissue derived mesenchymal stem cells and platelet concentrates: their association in vitro and in vivo. Vet Res Commun (2008) 32:51–5. 10.1007/s11259-008-9093-3 18683070

[28] Godwin EE Young NJ Dudhia J Beamish IC Smith RKW. Implantation of bone marrow-derived mesenchymal stem cells demonstrates improved outcome in horses with overstrain injury of the superficial digital flexor tendon. Equine Vet J (2012) 44:25–32. 10.1111/j.2042-3306.2011.00363.x 21615465

[29] Dyson SJ. Medical management of superficial digital flexor tendonitis: a comparative study in 219 horses (1992-2000). Equine Vet J (2004) 36:415–9. 10.2746/0425164044868422 15253082

[30] Guest DJ Smith MRW Allen WR. Monitoring the fate of autologous and allogeneic mesenchymal progenitor cells injected into the superficial digital flexor tendon of horses: preliminary study. Equine Vet J (2008) 40:178–81. 10.2746/042516408X276942 18267891

[31] Carrade DD Owens SD Galuppo LD Vidal MA Ferraro GL Librach F Clinicopathologic findings following intra-articular injection of autologous and allogeneic placentally derived equine mesenchymal stem cells in horses. Cytotherapy (2011) 13:419–30. 10.3109/14653249.2010.536213 21105841

[32] Carrade DD Lame MW Kent MS Clark KC Walker NJ Borjesson DL Comparative analysis of the immunomodulatory properties of equine adult-derived mesenchymal stem cells. Cell Med (2012) 4:1–11. 10.3727/215517912X647217 23152950 PMC3495591

[33] Carrade DD Borjesson DL. Immunomodulation by mesenchymal stem cells in veterinary species. Comp Med (2013) 63:207–17. 23759523 PMC3690426

[34] Kol A Wood JA Carrade Holt DD Gillette JA Bohannon-Worsley LK Puchalski SM Multiple intravenous injections of allogeneic equine mesenchymal stem cells do not induce a systemic inflammatory response but do alter lymphocyte subsets in healthy horses. Stem Cell Res Ther (2015) 6:73. 10.1186/s13287-015-0050-0 25888916 PMC4446064

[35] Broeckx S Borena BM Zimmerman M Mariën T Seys B Suls M Intravenous application of allogenic peripheral blood-derived mesenchymal stem cells: a safety assessment in 291 equine recipients. Curr Stem Cell Res Ther (2014) 9:452–7. 10.2174/1574888X09666140220003847 24548143

[36] Joswig AJ Mitchell A Cummings KJ Levine GJ Gregory CA Smith R Repeated intra-articular injection of allogeneic mesenchymal stem cells causes an adverse response compared to autologous cells in the equine model. Stem Cell Res Ther (2017) 8:42. 10.1186/s13287-017-0503-8 28241885 PMC5329965

[37] Caron JP Osteoarthritis. In: Ross MW Dyson SJ, editors. Diagnosis and Management of Lameness in the Horse. St. Louis, MO: Elsevier Saunders (2011). 666 p.

[38] Ardenaz N Vazquez FJ Romero A Remacha AR Barrachina L Sanz A Inflammatory response to the administration of mesenchymal stem cells in an equine experimental model: effect of autologous, and single and repeat doses of pooled allogeneic cells in healthy joints. Vet Res (2016) 12:65 10.1186/s12917-016-0692-x PMC4815220 27029614

[39] Owens SD Kol A Walker NJ Borjesson D. Allogeneic mesenchymal stem cell treatment induces specific alloantibodies in horses. Stem Cells Int (2016) 2016:5830103. 10.1155/2016/5830103 27648075 PMC5018342

[40] Berglund AK Schnabel LV. Allogeneic major histocompatibility complex-mismatched equine bone marrow-derived mesenchymal stem cells are targeted for death by cytotoxic anti-major histocompatibility complex antibodies. Equine Vet J (2017) 49:539–44. 10.1111/evj.12647 27862236 PMC5425313

[41] Pezzanite LM Fortier LA Antczak DF Cassano JM Brosnahan MM Miller D Equine allogeneic bone marrow-derived mesenchymal stromal cells elicit antibody responses in vivo. Stem Cell Res Ther (2015) 6:54. 10.1186/s13287-015-0053-x 25889095 PMC4414005

[42] Berglund AK Fischer MB Cameron KA Poole EJ Schnabel LV Transforming growth factor-β2 downregulates major histocompatibility complex (MHC) I and MHC II surface expression on equine bone marrow-derived mesenchymal stem cells without altering other phenotypic cell surface markers. Front Vet Sci (2017) 4:84 10.3389/fvets.2017.00084 28660198 PMC5466990

[43] Boswell SG Schnabel LV Mohammed HO Sundman EA Minas T Fortier LA. Increasing platelet concentrations in leukocyte-reduced platelet-rich plasma decrease collagen gene synthesis in tendons. Am J Sports Med (2014) 42:42–9. 10.1177/0363546513507566 24136860

[44] McCarrel TM Minas T Fortier LA. Optimization of leukocyte concentration in platelet-rich plasma for the treatment of tendinopathy. J Bone Joint Surg Am (2012) 3:e143. 10.2106/JBJS.L.00019 23032594

[45] Riboh JC Saltzman BM Yanke AB Fortier L Cole BJ. Effect of leukocyte concentration on the efficacy of platelet-rich plasma in the treatment of knee osteoarthritis. Am J Sports Med (2016) 44:792–800. 10.1177/0363546515580787 25925602

[46] Toricelli P Fini M Filardo G Tschon M Pischedda M Pacorini A Regenerative medicine for the treatment of musculoskeletal overuse injuries in competition horses. Int Orthop (2011) 35:1569–76. 10.1007/s00264-011-1237-3 21394594 PMC3174295

[47] Cowles RR Johnson LD Holoway PM Proximal suspensory desmitis: a retrospective study. Proceed Am Assoc Eq Pract (1994) 4:183–5.

[48] Bosch G van Weeren PR Barneveld A van Schie HTM Computerised analysis of standardised ultrasonographic images to monitor the repair of surgically created core lesions in equine superficial digital flexor tendons following treatment with intratendinous platelet rich plasma or placebo. Vet J (2011) 187:92–8. 10.1016/j.tvjl.2009.10.014 19932036

